# CT-based Hounsfield unit values reflect the degree of steatohepatitis in patients with low-grade fatty liver disease

**DOI:** 10.1186/s12876-023-02717-3

**Published:** 2023-03-17

**Authors:** Ha Neul Kim, Hong Jae Jeon, Hei Gwon Choi, In Sun Kwon, Woo Sun Rou, Jeong Eun Lee, Tae Hee Lee, Seok Hyun Kim, Byung Seok Lee, Kyung Sook Shin, Hyun Jung Lee, Hyuk Soo Eun

**Affiliations:** 1 Department of Medical Sciences, Chungnam National University, 266 Munwha-Ro, Jung-Gu, Daejeon, 35015 Republic of Korea; 2 Brain Korea 21 FOUR Project for Medical Science, Chungnam National University, 266 Munwha-ro, Jung-gu, Daejeon, 35015 Republic of Korea; 3 Department of Internal Medicine, Chungnam National University Sejong Hospital, 20, Bodeum 7-Ro, Sejong, 30099 Republic of Korea; 4 Department of Internal Medicine, Chungnam National University School of Medicine, 266 Munwha-Ro, Jung-Gu, Daejeon, 35015 Republic of Korea; 5 Research Institute of Medical Sciences, Chungnam National University School of Medicine, 266 Munwha-Ro, Jung-Gu, Daejeon, 35015 Republic of Korea; 6 Statistical Consultation of Clinical Trials Center, Chungnam National University Hospital, 266 Munwha-Ro, Jung-Gu, Daejeon, 35015 Republic of Korea; 7 Department of Radiology, Chungnam National University Hospital, 282 Munwha-Ro, Jung-Gu, Daejeon, 35015 Republic of Korea; 8 Department of Radiology, Chungnam National University School of Medicine, 266 Munwha-Ro, Jung-Gu, Daejeon, 35015 Republic of Korea; 9 Department of Biomedical Laboratory Science, Daegu Health College, Chang-Ui Building, 15 Yeongsong-Ro, Buk-Gu, Daegu, 41453 Republic of Korea; 10 Department of Internal Medicine, Chungnam National University Hospital, 282 Munwha-Ro, Jung-Gu, Daejeon, 35015 Republic of Korea; 11 Department of Pathology, Chungnam National University Hospital, 282 Munwha-Ro, Jung-Gu, Daejeon, 35015 Republic of Korea; 12 Department of Pathology, Chungnam National University School of Medicine, 266 Munwha-Ro, Jung-Gu, Daejeon, 35015 Republic of Korea

**Keywords:** Nonalcoholic fatty liver disease, Hepatic steatosis, Nonalcoholic steatohepatitis, Hounsfield unit, Liver biopsy

## Abstract

**Background/Aims:**

Nonalcoholic fatty liver disease (NAFLD) is the most common liver disease worldwide. Ultrasound, the most used tool for diagnosing NAFLD, is operator-dependent and shows suboptimal performance in patients with mild steatosis. However, few studies have been conducted on whether alternative noninvasive methods are useful for diagnosing mild hepatic steatosis. Also, little is known about whether noninvasive tests are useful for grading the severity of hepatic steatosis or the degree of intrahepatic inflammation. Therefore, we aimed to evaluate whether the HSI, the FLI and HU values in CT could be used to discriminate mild hepatic steatosis and to evaluate the severity of hepatic steatosis or the degree of intrahepatic inflammation in patients with low-grade fatty liver disease using liver biopsy as a reference standard.

**Methods:**

Demographic, laboratory, CT imaging, and histological data of patients who underwent liver resection or biopsy were analyzed. The performance of the HSI, HU values and the FLI for diagnosing mild hepatic steatosis was evaluated by calculating the area under the receiver operating characteristic curve. Whether the degree of hepatic steatosis and intrahepatic inflammation could be predicted using the HSI, HU values or the FLI was also analyzed. Moreover, we validate the results using magnetic resonance imaging proton density fat fraction as an another reference standard.

**Results:**

The AUROC for diagnosing mild hepatic steatosis was 0.810 (*p* < 0.001) for the HSI, 0.732 (*p* < 0.001) for liver HU value, 0.802 (*p* < 0.001) for the difference between liver and spleen HU value (L-S HU value) and 0.813 (*p* < 0.001) for the FLI. Liver HU and L-S HU values were negatively correlated with the percentage of hepatic steatosis and NAFLD activity score (NAS) and significantly different between steatosis grades and between NAS grades. The L–S HU value was demonstrated the good performance for grading the severity of hepatic steatosis and the degree of intrahepatic inflammation.

**Conclusions:**

The HU values on CT are feasible for stratifying hepatic fat content and evaluating the degree of intrahepatic inflammation, and the HSI and the FLI demonstrated good performance with high sensitivity and specificity in diagnosing mild hepatic steatosis.

**Supplementary Information:**

The online version contains supplementary material available at 10.1186/s12876-023-02717-3.

## Introduction

Nonalcoholic fatty liver disease (NAFLD) is the most common liver disease worldwide, characterized by an excessive accumulation of intrahepatic fat associated with insulin resistance [1]. Globally, the prevalence of NAFLD diagnosed by imaging tests is approximately 25.24% [2–4]. Patients diagnosed with nonalcoholic steatohepatitis (NASH), a progressive form of NAFLD which indicates hepatic inflammation and steatosis on histology, have an increased risk of progression of fibrosis, liver cirrhosis, and hepatocellular carcinoma [5–8].

Liver biopsy is the gold standard for diagnosing NAFLD. Based on the percentage of hepatocytes that contain fat vacuoles, steatosis is classified as normal or grade 0 if steatotic hepatocytes are < 5%; mild or grade 1 if 5%–33%; moderate or grade 2 if 34%–66%; and severe or grade 3 if > 66% [9–11]. However, liver biopsy is an invasive technique with potentially fatal complications [12]. Therefore, noninvasive methods for diagnosing fatty liver are preferred in various clinical settings. In particular, ultrasound is the most commonly used method. However, it is operator-dependent and has suboptimal performance in diagnosing mild steatosis and grading the severity of hepatic steatosis [13–16]. Therefore, CT is often used as an initial evaluation for patients with elevated liver enzyme levels and suspected hepatic steatosis, especially patients with obesity with poor sonic window on ultrasound examination. In many studies, both the liver HU value and the difference between the HU value of the liver and spleen (L–S HU value) have proven to be useful tools for diagnosing fatty liver disease [17–20]. Moreover, some guidelines recommend that scores using serum biomarkers could provide an alternative mean for diagnosing NAFLD [21]. Considering the under-diagnosis of and the lack of adequate care for NAFLD in the primary care setting, introducing an effective but simple steatosis scoring system that can be easily used by primary care providers is necessary [22, 23]. Among various steatosis scores, the hepatic steatosis index (HSI) and the fatty liver index (FLI) are ones of the simplest and consist of easily obtainable information [24]. HSI consists of sex, the presence of type 2 diabetes mellitus (DM), body mass index (BMI), and aspartate aminotransferase (AST) and alanine aminotransferase (ALT) levels, while fatty liver index consists of BMI, waist circumference, triglycerides (TG) level, and gamma-glutamyl transferase (GGT) level. However, studies which aimed to evaluate the usefulness of noninvasive methods, including CT, the HSI, and the FLI, in diagnosing mild hepatic steatosis, which is relatively common in clinical practice, are few. Additionally, whether the HU value, the HSI, or the FLI correlates with the histological severity of hepatic steatosis remains unclear.

Meanwhile, several guidelines have stated that these noninvasive methods have limitations in diagnosing steatohepatitis [2, 25]. The presence of steatohepatitis is known to be the most important factor in the progression of fibrosis, while the severity of fibrosis is the most important histologic marker associated with the incidence of liver-related complications and mortality in patients with NAFLD [26–29]. In addition, since improvement of intrahepatic inflammation is known to be associated with improvement of fibrosis, the improvement of intrahepatic inflammation has been used as surrogate endpoints in various clinical trials [30]. NAFLD activity score (NAS) is a widely used scoring system to evaluate the degree of steatohepatitis in patients with NAFLD [11]. NAS is based on histological findings and calculated by scoring the degree of steatosis, hepatocyte ballooning, and inflammation and summing these values. The usefulness of NAS has been confirmed in several studies and is recommended as a method for evaluating changes in liver histology in patients with NASH. However, since liver biopsy is invasive and carries the risk of complications, it has limitations in being used as a method for evaluating the improvement of steatohepatitis in a general clinical practice. While several guidelines have stated that noninvasive tests are not acceptable alternative to biopsy for the diagnosis of NASH, few studies have been conducted on whether noninvasive tests are useful for evaluating the severity of intrahepatic inflammation in patients with biopsy-proven NAFLD [2, 25]. If there is a noninvasive method that can evaluate the severity of intrahepatic inflammation in patients with biopsy-proven NAFLD, it will be useful for determining the effectiveness of treatment in clinical situations.

Therefore, we aimed to evaluate whether HU values, the HSI, and the FLI ​​could be helpful in diagnosing mild hepatic steatosis, stratifying the severity of hepatic steatosis and predicting inflammatory activity in patients with low-grade hepatic steatosis.

## Materials and methods

### Study design and participants

Patients aged between 18 and 75 years who underwent histological examination of the liver at Chungnam National University Hospital between January 2008 and December 2022 were enrolled in this study. Patients who underwent CT within 3 months prior to liver biopsy were included, and their electronic medical records were retrospectively reviewed. We excluded patients who had steatosis in > 33% of the hepatocytes on liver biopsy, therefore, only patients with mild or grade 1 hepatic steatosis were enrolled. We also enrolled patients with a magnetic resonance imaging proton density fat fraction (MRI-PDFF) of less than 33% among patients who underwent MRI-PDFF and CT within 6 months of MRI-PDFF at Chungnam National University Hospital. Patients with other liver diseases, such as chronic viral hepatitis B, chronic viral hepatitis C, autoimmune hepatitis, and primary biliary cholangitis, and those with excessive alcohol consumption (≥ 30 g/d of alcohol consumption for men and ≥ 20 g/d for women) were excluded. Additionally, patients with liver cirrhosis and a history of hepatocellular carcinoma or other liver-related malignancies within 5 years were also excluded.

### Data collection, calculation of the HSI and the FLI, and measurement of the HU value

We reviewed the histological data of the enrolled patients and collected demographic, laboratory, CT imaging, and MRI-PDFF data by investigating electronic medical records. From the data collected, the HSI and the FLI were calculated. The HSI was calculated as ‘8 × (ALT/AST ratio) + BMI (+ 2, if female; + 2, if with DM)’ [31], while the FLI was calculated as '(e^0.953 × ln(TG) + 0.139 × BMI + 0.718 × ln(GGT) + 0.053 × WC −15.745^/ 1 + e^0.953 × ln(TG) + 0.139 × BMI + 0.718 × ln(GGT) + 0.053 × WC −15.754^) × 100'. We also used only pre-contrast CT images to measure the HU values of the liver and spleen. Specifically, the HU values of ten randomly selected parts of the liver and spleen were measured, and the averages of HU values were calculated. The area measured at each time was set to 2.5–3 cm^2^. We defined the average of HU values of the liver as the liver HU value. The L–S HU value was calculated by subtracting the average HU value of the spleen from the average HU value of the liver.

### Definition of hepatic steatosis, low-grade hepatic steatosis, mild steatosis grade, and NAS grade

Hepatic steatosis was defined as the accumulation of fat vacuoles in > 5% of hepatocytes. In our study, low-grade hepatic steatosis was defined as the presence of steatosis in < 33% of hepatocytes, and these patients were classified again according to the percentage of hepatic steatosis as follows: steatosis < 5%, the mild steatosis grade 0 (mild G0 or mG0) group; steatosis 5%–19%, the mild steatosis grade 1 (mild G1 or mG1) group; and steatosis 20%–33%, the mild steatosis grade 2 (mild G2 or mG2) group. Similarly, patients who underwent MRI-PDFF were classified according to MRI-PDFF as follows: MRI-PDFF < 5%, the mild steatosis grade 0 (mild G0 or mG0) group; MRI-PDFF 5%–19%, the mild steatosis grade 1 (mild G1 or mG1) group; and MRI-PDFF 20%–33%, the mild steatosis grade 2 (mild G2 or mG2) group. In our study, each NAS grade was defined as follows. NAS < 3, the grade 1 (G1); NAS 3–4, grade 2 (G2); and NAS > 4, grade 3 (G3).

### Statistical analyses

The Student’s t-test for continuous data and chi-squared test for categorical data were used to compare the baseline characteristics between patients with and without hepatic steatosis. We evaluated the performance of each method to diagnose hepatic steatosis by calculating the area under the receiver operating characteristic curve (AUROC). Correlations between variables were determined using Pearson correlation coefficient. Logistic regression analyses were performed to identify independent predictive factors of hepatic steatosis. All factors with a *p* < 0.05 in the univariate analysis were included in the multivariate analysis, with the exception of multivariate analysis to assess whether the HSI or the FLI are an independent predictive factor for hepatic steatosis. In that exceptional case, DM, BMI, and the AST and ALT levels were excluded for calculating the HSI and BMI, waist circumference, TG level, and GGT level were excluded for calculating the FLI due to potential multicollinearity. Student’s t-test was used to compare the HSI, liver HU value, L-S HU value and the FLI between the two steatosis grade groups or the two NAS grade groups. All statistical analyses were performed using SPSS (version 26.0; IBM Corp., Armonk, NY, USA).

## Results

**Fig. 1:**
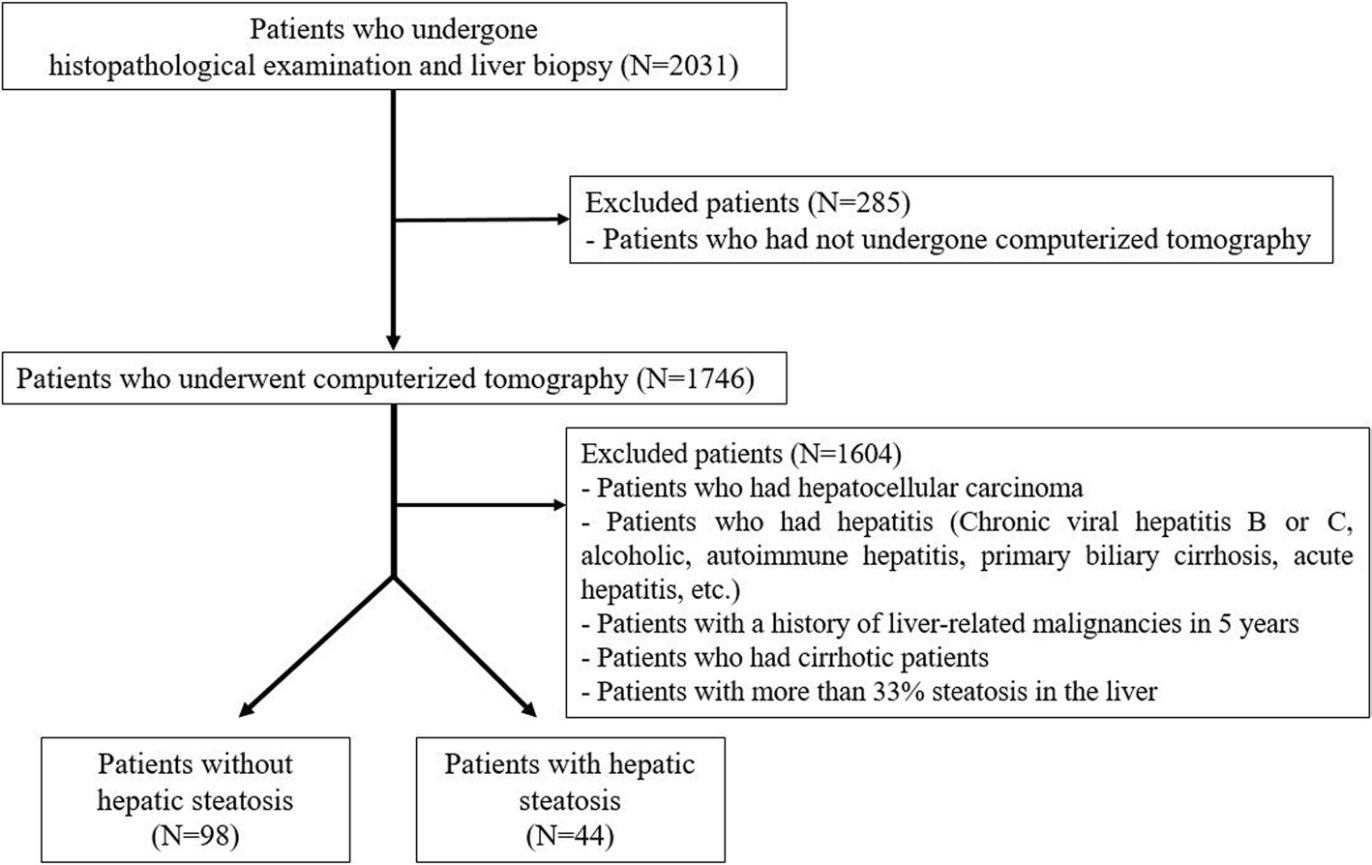
Flow chart showing enrollment of patients who underwent liver biopsy or liver resection

Altogether, the data of 2,031 patients aged between 18 and 75 years, who underwent liver biopsy or hepatic resection at Chungnam National University Hospital between January 2008 and December 2022, were reviewed. Among them, 1,746 patients underwent CT within 3 months prior to liver biopsy or hepatic resection, and the rest 285 patients were excluded. And among these 1,746 patients, 1,604 patients who did not meet the enroll criteria were sequentially excluded. Finally, of the patients who underwent liver biopsy or liver resection, 142 patients were enrolled in our study. Of the 142 patients analyzed, 44 had hepatic steatosis ≥ 5%, and 98 patients had hepatic steatosis < 5% or did not have clinically significant hepatic steatosis (Fig. 1).

### Comparison of the baseline characteristics of patients with and without hepatic steatosis

The baseline characteristics of the patients are summarized in Table 1. The number of patients classified into the mild steatosis group was 98 (mG0), 33 (mG1), 11 (mG2). And the number of patients classified into the steatohepatitis group using NAFLD activity score (NAS) was 23 (G1), 29 (G2), 6 (G3). BMI and serum AST, ALT, triglyceride (TG), albumin levels, and waist circumference (WC) were significantly higher in patients with hepatic steatosis than in those without hepatic steatosis. The mean HSI value was higher in patients with hepatic steatosis (37.37, 95% confidence interval [CI]: 35.60–39.31) than in those without hepatic steatosis (31.54, 95% CI: 30.62–32.88) (*p* < 0.001), while mean liver HU value and mean L–S HU value were lower in patients with hepatic steatosis (liver HU: 46.56, 95% CI: 45.85–51.75/L–S HU: -1.401, 95% CI: -2.586–3.051) than in those without hepatic steatosis (liver HU: 54.38 95% CI: 53.74–56.63/L–S HU: 7.813, 95% CI: 6.884–9.936) (both *p* < 0.001). The mean FLI value was higher in patients with hepatic steatosis (61.83, 95% CI: 54.07–69.55) than in those without hepatic steatosis (33.06, 95% CI: 27.55–38.89) (*p* < 0.001).

**Table 1:** A comparison of characteristics between participants with and without hepatic steatosis

Baseline characteristics			
Variables	**Steatosis (–)** **(*N* = 98)**	**Steatosis ( +)** **(*N* = 44)**	*p* value
Demographic variables			
Age (years)	54.53 ± 13.85	44.93 ± 14.66	< 0.001
Gender (M/F)	34/64	22/22	
Body mass index (kg/m^2^)	23.05 ± 3.243	26.26 ± 3.493	< 0.001
Comorbidities			
Diabetes mellitus	11 (11.2%)	10 (22.7%)	
Hypertension	11 (11.2%)	5 (11.4%)	
Dyslipidemia	13 (13.3%)	3 (6.82%)	
Biochemical parameters			
Aspartate aminotransferase (IU/L)	28.56 ± 19.72	41.55 ± 33.44	0.004
Alanine aminotransferase (IU/L)	26.47 ± 23.40	53.02 ± 65.72	0.001
Triglycerides (mg/dL)	103.6 ± 62.79	202.4 ± 124.9	< 0.001
Total cholesterol (mg/dL)	184.2 ± 46.02	200.8 ± 47.92	0.068
Total bilirubin (mg/dL)	0.970 ± 0.985	0.932 ± 0.973	0.830
Gamma-glutamyl transpeptidase (IU/L)	119.9 ± 158.0	111.8 ± 133.8	0.7720
Serum glucose (mg/dL)	105.9 ± 36.69	119.3 ± 45.89	0.064
Serum albumin (g/dL)	3.812 ± 0.580	4.161 ± 0.500	< 0.001
Platelet count (10^3^/uL)	247.9 ± 83.54	259.9 ± 81.19	0.419
Waist circumference (cm)	81.65 ± 9.249	91.64 ± 9.737	< 0.001
Liver histology			
Steatosis grade S0/S1/S2/S3	98/0/0/0	8/36/0/0	
Mild steatosis grade mG0/mG1/mG2	98/0/0	0/33/11	
METAVIR score F0/F1/F2/F3/F4	76/15/6/1/0	28/7/6/3/0	
NAFLD activity score grade G1/G2/G3	16/0/0	7/29/6	
Hepatic steatosis index (mean ± SD)	31.54 ± 4.090	37.37 ± 5.729	< 0.001
Liver HU (mean ± SD)	54.38 ± 6.125	46.56 ± 9.911	< 0.001
Liver HU-Spleen HU (mean ± SD)	7.813 ± 6.198	-1.401 ± 8.988	< 0.001
Fatty liver index (mean ± SD)	33.06 ± 22.97	61.83 ± 21.74	< 0.001

### Comparison of performance of the HSI, liver HU value, L–S HU value and the FLI for diagnosing mild hepatic steatosis

**Fig. 2:**
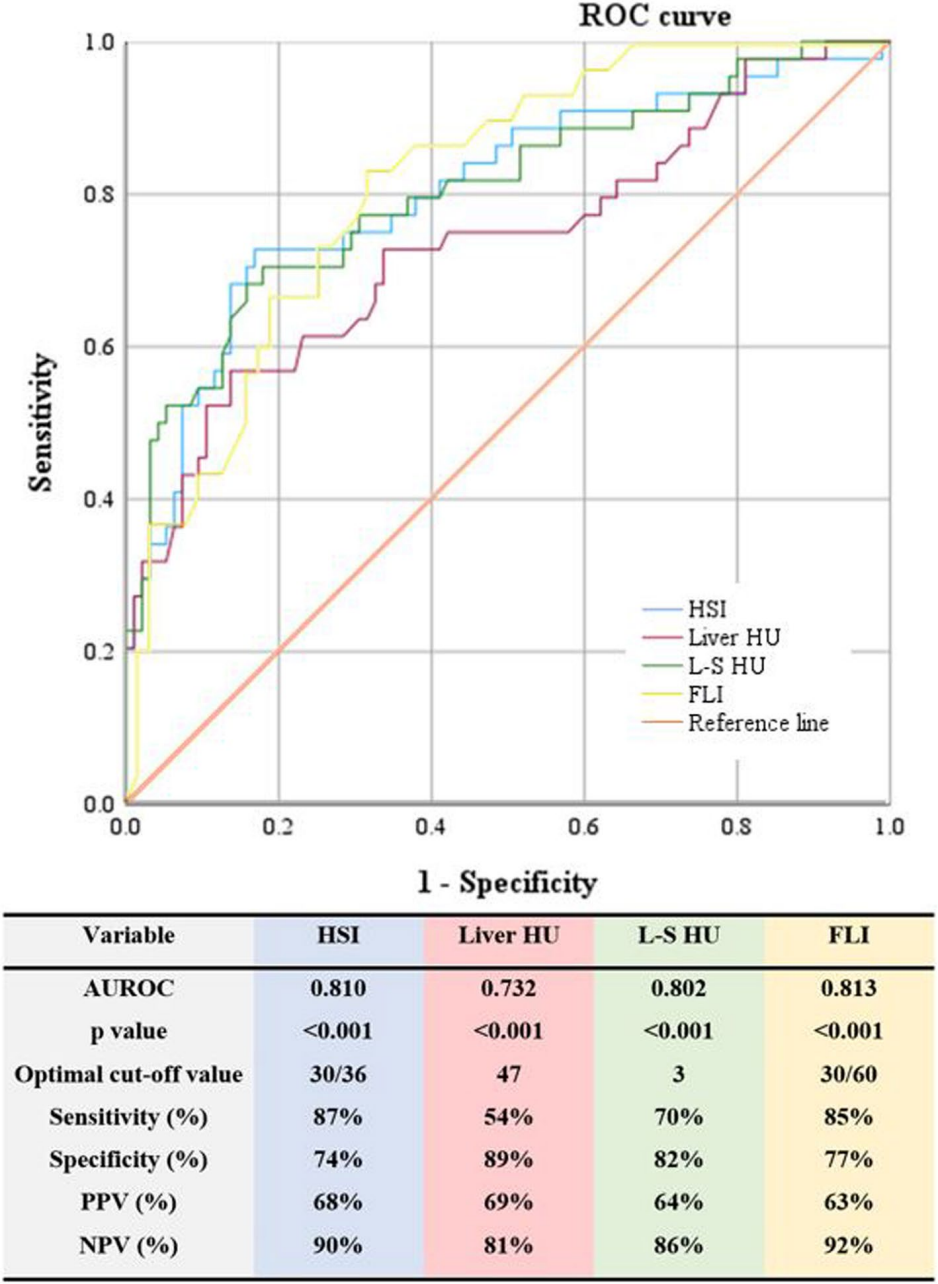
ROC curves and diagnostic performance of hepatic steatosis index, liver HU value, liver HU value-spleen HU value and fatty liver index for diagnosing mild hepatic steatosis

The HSI had the highest AUROC for diagnosing hepatic steatosis (AUROC 0.810), followed by L–S HU value (AUROC 0.802), liver HU value (AUROC 0.732) and the FLI (AUROC 0.813) (Fig. 2). The HSI, with a low cut-off value of 30 and a high cut-off value of 36, diagnosed hepatic steatosis with 87% sensitivity and 74% specificity. Additionally, the L–S HU value with a cut-off value of 3 diagnosed hepatic steatosis with 70% sensitivity and 82% specificity, while the liver HU value with a cut-off value of 47 diagnosed hepatic steatosis with 54% sensitivity and 89% specificity. The FLI, with a low cut-off value of 30 and a high cut-off value of 60, diagnosed hepatic steatosis with 85% sensitivity and 77% specificity.

### Factors associated with hepatic steatosis

The univariate analysis revealed that age, BMI, serum AST, ALT, TG, albumin levels, WC, the HSI, liver HU value, L–S HU value and the FLI were associated with hepatic steatosis. In the multivariate analysis, HSI, L–S HU value and the FLI remained as independent diagnostic factors for hepatic steatosis (Tables 2, 3 and 4). In patients with hepatic steatosis, the liver HU value was negatively correlated with BMI, AST, ALT, TG and glucose level. The L–S HU value was also negatively correlated with BMI, AST, ALT, TG and glucose level in patients with hepatic steatosis (data not shown).

**Table 2:** Univariate and multivariate analyses using the Hepatic steatosis index for patients with and without hepatic steatosis

	Univariate analysis		Multivariate analysis	
Variables	OR (95% CI)	*p* value	OR (95% CI)	*p* value
Age	0.96(0.93–0.98)	0.001	0.99(0.94–1.04)	0.636
Hypertension	0.90(0.29–2.80)	0.851		
Hyperlipidemia	2.28(0.62–8.37)	0.215		
HSI	1.31(1.18–1.45)	< 0.001	1.32(1.09–1.61)	0.005
TG (mg/dL)	1.01(1.01–1.02)	< 0.001	1.01(1.00–1.02)	0.037
TC (mg/dL)	1.01(1.00–1.02)	0.067		
TB (mg/dL)	0.96(0.65–1.41)	0.830		
GGT (U/L)	1.00(1.00–1.00)	0.781		
Glucose (mg/dL)	1.01(1.00–1.02)	0.076		
Albumin (g/dL)	4.79(1.89–12.1)	0.001	14.3(1.79–113.3)	0.012
Platelets(10^3^/μL)	1.00(1.00–1.01)	0.422		
Waist circumference (cm)	1.12(1.07–1.17)	< 0.001	1.00(0.93–1.08)	0.933

Multivariate analysis adjusted model: Diabetes, *BMI* Body mass index, *AST* Aspartate aminotransferase, *ALT* Alanine aminotransferase, were excluded because they were correlated with HSI

*CI* Confidence interval, *HU* Hounsfield unit

**Table 3:** Univariate and multivariate analyses using the liver-spleen Hounsfield unit for patients with and without hepatic steatosis

	Univariate analysis		Multivariate analysis	
Variables	OR (95% CI)	*p* value	OR (95% CI)	*p* value
Age	0.96(0.93–0.98)	0.001	0.99(0.94–1.04)	0.712
Diabetes	0.44(0.17–1.12)	0.084		
Hypertension	0.90(0.29–2.80)	0.851		
Hyperlipidemia	2.28(0.62–8.37)	0.215		
L-S HU	0.84(0.78–0.90)	< 0.001	0.84(0.74–0.96)	0.011
BMI	1.33(1.17–1.50)	< 0.001	1.07(0.77–1.47)	0.690
AST (U/L)	1.02(1.01–1.04)	0.010	0.99(0.95–1.04)	0.779
ALT (U/L)	1.02(1.01–1.04)	0.003	1.02(0.98–1.07)	0.333
TG (mg/dL)	1.01(1.01–1.02)	< 0.001	1.01(1.00–1.02)	0.041
TC (mg/dL)	1.01(1.00–1.02)	0.067		
TB (mg/dL)	0.96(0.65–1.41)	0.830		
GGT (U/L)	1.00(1.00–1.00)	0.781		
Glucose (mg/dL)	1.01(1.00–1.02)	0.076		
Albumin (g/dL)	4.79(1.89–12.1)	0.001	17.2(2.07–142.1)	0.008
Platelets (10^3^/μL)	1.00(1.00–1.01)	0.422		
Waist circumference (cm)	1.12(1.07–1.17)	< 0.001	1.01(0.91–1.13)	0.844

*CI* Confidence interval, *HU* Hounsfield unit

**Table 4:** Univariate and multivariate analyses using the Fatty liver index for patients with and without hepatic steatosis

	Univariate analysis		Multivariate analysis	
Variables	OR (95% CI)	*p* value	OR (95% CI)	*p* value
Age	0.96(0.93–0.98)	0.001	0.96(0.91–1.02)	0.209
Diabetes	0.44(0.17–1.12)	0.084		
Hypertension	0.90(0.29–2.80)	0.851		
Hyperlipidemia	2.28(0.62–8.37)	0.215		
FLI	1.05(1.03–1.08)	< 0.001	1.08(1.03–1.13)	0.002
AST (U/L)	1.02(1.01–1.04)	0.010	1.00(0.95–1.05)	0.911
ALT (U/L)	1.02(1.01–1.04)	0.003	1.02(0.98–1.07)	0.334
TC (mg/dL)	1.01(1.00–1.02)	0.067		
TB (mg/dL)	0.96(0.65–1.41)	0.830		
Glucose (mg/dL)	1.01(1.00–1.02)	0.076		
Albumin (g/dL)	4.79(1.89–12.1)	0.001	97.5(6.80–1397.3)	0.001
Platelets (10^3^/μL)	1.00(1.00–1.01)	0.422		
Waist circumference (cm)	1.12(1.07–1.17)	< 0.001	0.96(0.87–1.06)	0.447

*CI* Confidence interval, *HU* Hounsfield unit

Multivariate analysis adjusted model: *BMI* Body mass index, *TG* Triglycerides, *GGT* Gamma-glutamyl transpeptidase, were excluded because they were correlated with FLI

### Distribution and performance of the HSI, liver HU value, L–S HU value and FLI according to steatosis grade group

**Fig. 3:**
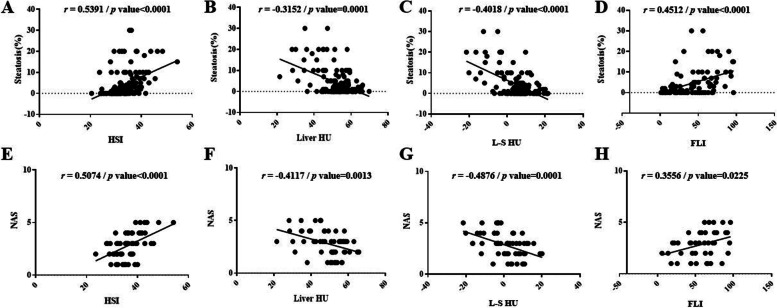
The correlation between each index and the percentage of hepatic steatosis. **A** Scatter plots showing the positive correlation between the hepatic steatosis index and the percentage of hepatic steatosis (**B**) Scatter plots showing the negative correlation between liver HU value and the percentage of hepatic steatosis (**C**) Scatter plots showing the negative correlation between liver HU value-spleen HU value and the percentage of hepatic steatosis (**D**) Scatter plots showing the positive correlation between the fatty liver index and the percentage of hepatic steatosis (**E**) Scatter plots showing the positive correlation between the hepatic steatosis index and the NAFLD activity score (**F**) Scatter plots showing the negative correlation between liver HU value and the NAFLD activity score (**G**) Scatter plots showing the negative correlation between liver HU value-spleen HU value and the NAFLD activity score (**H**) Scatter plots showing the positive correlation between the fatty liver index the NAFLD activity score

**Fig. 4:**
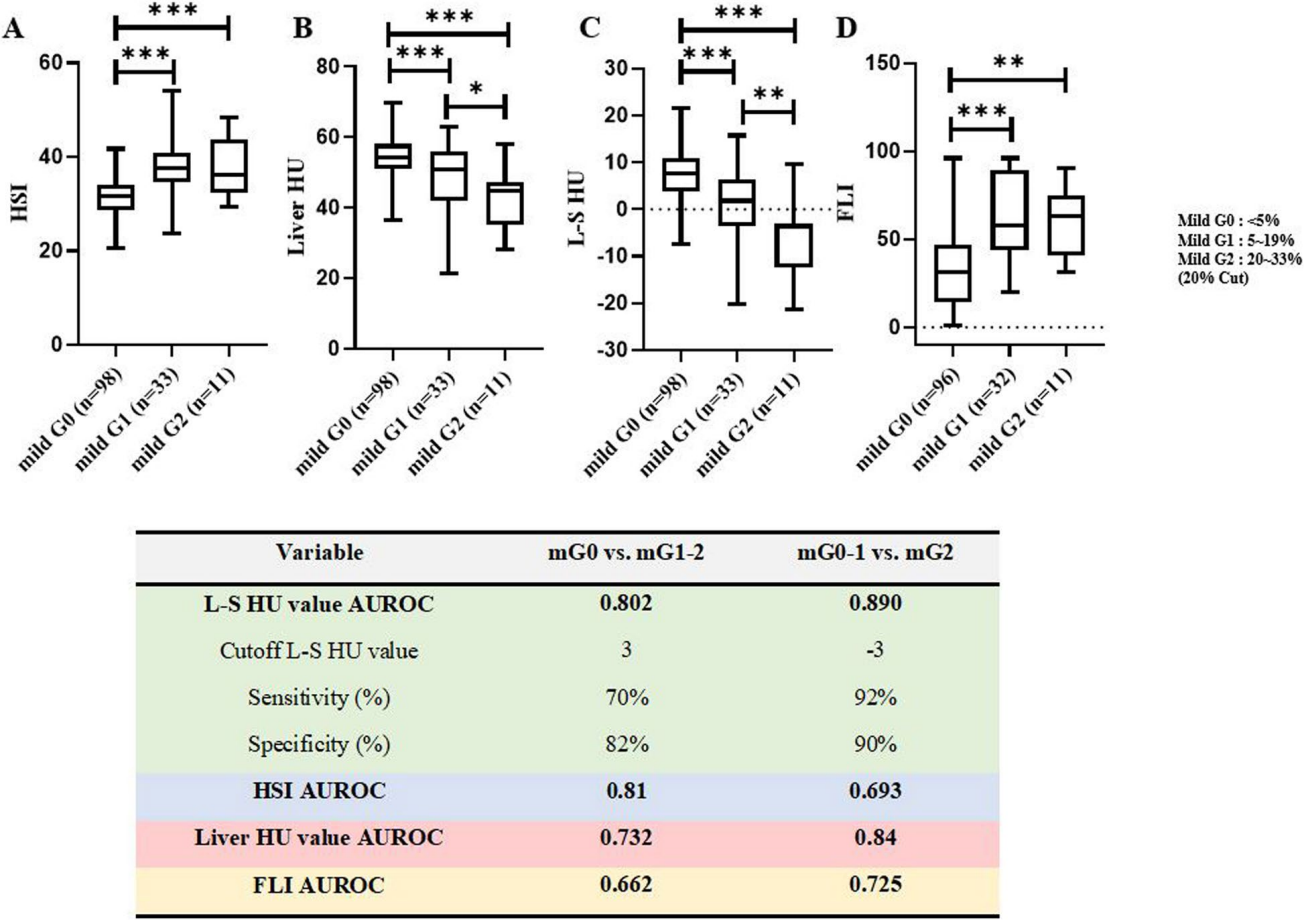
The comparison of each index according to steatosis grade group and performance of each index in grading the severity of hepatic steatosis. **A** The comparison of hepatic steatosis index according to mild steatosis grade group. **B** The comparison of liver HU value according to mild steatosis grade group. **C** The comparison of liver HU value-spleen HU value according to mild steatosis grade group. **D** The comparison of fatty liver index according to mild steatosis grade group. Performance of hepatic steatosis index, liver HU value, liver HU value-spleen HU value and fatty liver index in grading the severity of hepatic steatosis was also shown. Mild G0 = Group consisting of patients with the percentage of hepatic steatosis < 5%; mild G1 = Group consisting of patients with the percentage of hepatic steatosis of ≥ 5% and < 20%; mild G2 = Group consisting of patients with the percentage of hepatic steatosis ≥ 20% and < 33%

The percentage of hepatic steatosis was positively correlated with the HSI (*r* = 0.5391) (*p* < 0.0001) or the FLI (*r* = 0.4512) (*p* < 0.0001), and negatively correlated with liver HU value (*r* =  − 0.3152) (*p* = 0.0001) or L–S HU value (*r* =  − 0.4018) (*p* < 0.0001) (Fig. 3). The mean liver HU value for patients in mG0, mG1, and mG2 was 55.2 (95% CI: 55.15–55.25), 49.49 (95% CI: 49.38–49.60), and 46.93 (95% CI: 46.78–47.08), respectively. Liver HU value was significantly different between mG0 and mG1(*p* < 0.001) or mG2 (*p* < 0.001) and between mG1 and mG2 (*p* = 0.04) (Fig. 4). Moreover, the mean L–S HU values for patients in mG0, mG1, and mG2 were 8.497 (95% CI: 8.449–8.545), 1.292 (95% CI: 1.190–1.394), and -3.024 (95% CI: -3.166–-2.883), respectively. The L–S HU value was also significantly different between mG0 and mG1(*p* < 0.001) or mG2 (*p* < 0.001) and between mG1 and mG2 (*p* = 0.01). Although the HSI was also significantly different between mG0 and mG1 (*p* < 0.001) or mG2 (*p* < 0.001), the differences between mG1 and mG2 were not statistically significant (*p* = 0.47). The FLI was significantly different between mG0 and mG1 (*p* < 0.001) or mG2 (*p* = 0.016), the differences between mG1 and mG2 were not statistically significant (*p* = 0.43).

### Performance of the HSI, liver HU value, L-S HU value and the FLI in grading the severity of hepatic steatosis

Figure 4 shows the AUROCs of the HSI, liver HU value, L–S HU value and the FLI for grading the severity of hepatic steatosis. The L–S HU value demonstrated the best performance in grading the severity of low-grade hepatic steatosis. The optimal cut-off L–S HU values were 3 HU for ≥ mG1, and -3 HU for ≥ mG2.

### Distribution and performance of the HSI, liver HU value, L–S HU value and the FLI according to NAS grade group

**Fig. 5:**
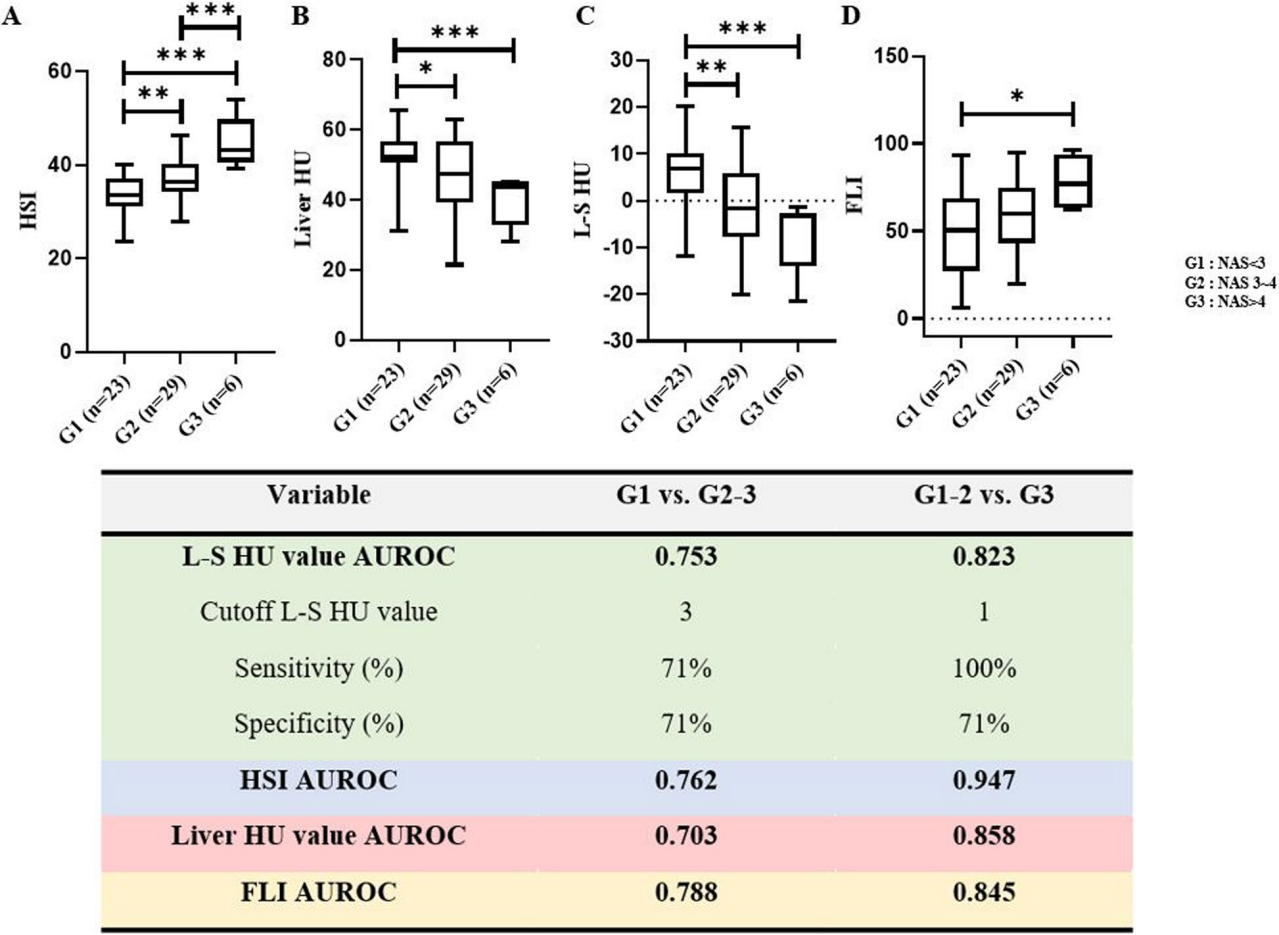
The comparison of each index according to NAFLD activity score group and performance of each index in grading the severity of steatohepatitis. **A** The comparison of hepatic steatosis index according to NAFLD activity score group. **B** The comparison of liver HU value according to NAFLD activity score group. **C** The comparison of liver HU value-spleen HU value according to NAFLD activity score group. **D** The comparison of fatty liver index according to NAFLD activity score group. Performance of hepatic steatosis index, liver HU value, liver HU value-spleen HU value and fatty liver index in grading the severity of steatohepatitis was also shown. G1 = Group consisting of patients with the NAFLD activity score < 3; G2 = Group consisting of patients with the NAFLD activity score 3–4; G3 = Group consisting of patients with the NAFLD activity score > 4

The NAS was positively correlated with the HSI (*r* = 0.5074) (*p* < 0.0001) or FLI (*r* = 0.3556) (*p* < 0.0001), and negatively correlated with liver HU value (*r* =  − 0.4117) (*p* = 0.0013) or L–S HU value (*r* =  − 0.4876) (*p* = 0.0001) (Fig. 3). The mean liver HU value for patients in G1, G2, and G3 was 52.49 (95% CI: 49.25–55.73), 47.13 (95% CI: 43.06–51.19), and 40.04 (95% CI: 32.56–47.51), respectively. Liver HU value was significantly different between G1 and G2 (*p* = 0.02) or G3 (*p* < 0.001) (Fig. 5). Moreover, the mean L–S HU values for patients in G1, G2, and G3 were 5.767 (95% CI: 2.632–8.902), -1.060 (95% CI: -4.714–2.593), and -7.35 (95% CI: -15.48–0.781), respectively. The L–S HU value was also significantly different between G1 and G2 (*p* = 0.003) or G3 (*p* < 0.001). The HSI was significantly different between G1 and G2(*p* = 0.004) or G3 (*p* < 0.001), and between G2 and G3 (*p* < 0.001). The FLI was significantly different between G1 and G3 (*p* = 0.02).

### Performance of the HSI, liver HU value, L-S HU value and the FLI in evaluating the degree of steatohepatitis

Figure 5 shows the AUROCs of the HSI, liver HU value, L–S HU value and the FLI for evaluating the degree of steatohepatitis. The L–S HU value, with a cut-off value of -3, predicted whether NAS was 3 or higher or not with 71% sensitivity and 71% specificity. And the L–S HU value, with a cut-off value of -1, predicted whether NAS was 5 or higher or not with 100% sensitivity and 71% specificity. Additionally, the HSI and the FLI had high AUROC for predicting NAS of 3 or more and NAS of 5 or more.

### Comparison of NAS between patients with and without metabolic syndrome

Among 2,031 patients aged between 18 and 75 years, who underwent liver biopsy or hepatic resection at Chungnam National University Hospital during study period, 285 patients who didn’t underwent CT within 3 months prior to liver biopsy or hepatic resection were excluded (Fig. S1). And among these 1,746 patients, 1,604 patients who did not meet the enroll criteria were sequentially excluded. Metabolic syndrome could not be evaluated in 21 patients of 142 patients due to missing variables. Among 77 patients without metabolic syndrome, hepatic steatosis was observed in 17 patients (22.1%), and among 44 patients with metabolic syndrome, hepatic steatosis was observed in 21 patients (47.8%) (*p* = 0.03). NAS was evaluated in 52 patients with steatotic hepatocytes on liver biopsy and there was no difference in NAS between the group with and without metabolic syndrome (*p* = 0.351).

### Distribution of the liver HU value and L–S HU value according to MRI-PDFF

During the study period, 152 patients underwent MRI-PDFF at Chungnam National University Hospital, and 88 of them underwent CT within 6 months. Of these 88 patients, 64 did not meet the enroll criteria. Therefore, of the patients who underwent MRI-PDFF, 22 patients were enrolled in our study. Of the 22 patients analyzed, 13 had MRI-PDFF ≥ 5%, and 9 patients had MRI-PDFF < 5% (Fig. S2). The percentage of hepatic steatosis was positively correlated with the HSI (*r* = 0.6794) (*p* = 0.0007) or the FLI (*r* = 0.6720) (*p* = 0.0030), and negatively correlated with liver HU value (*r* =  − 0.7638) (*p* < 0.0001) or L–S HU value (*r* =  − 0.5781) (*p* = 0.0024) (Fig. S3). The mean liver HU value for patients in mG0, mG1, and mG2 was 53.57 (95% CI: 47.27–59.86), 42.77 (95% CI: 36.55–48.99), and 29.80 (95% CI: 26.05–33.55), respectively. Liver HU value was significantly different between mG0 and mG1 (*p* = 0.006) or mG2 (*p* < 0.001) and between mG1 and mG2 (*p* = 0.014) (Fig. S4). Moreover, the mean L–S HU values for patients in mG0, mG1, and mG2 were 5.47 (95% CI: -0.62–11.6), -2.66 (95% CI: -8.75–3.43), and -10.8 (95% CI: -51.4–29.7), respectively. The L–S HU value was also significantly different between mG0 and mG1 (*p* = 0.02) and between mG0 and mG2 (*p* = 0.02). Although the HSI was significantly different between mG2 and mG0 (*p* < 0.001) or mG1 (*p* < 0.001). The FLI was also significantly different between mG0 and mG1 (*p* = 0.03) and between mG0 and mG2 (*p* = 0.03).

## Discussion

In our study, the HU values on CT were useful in quantifying and stratifying liver fat contents in patients with low-grade hepatic steatosis, and the HSI and the FLI was demonstrated good performance with high sensitivity and specificity in diagnosing mild hepatic steatosis. In addition, the HU values were useful in evaluating the degree of intrahepatic inflammation in patients with low-grade hepatic steatosis. Transabdominal ultrasound, which is a commonly used diagnostic test for fatty liver disease in clinical field, has various limitations, such as a poor sonic window in patients with obesity and subjectivity according to the operator, resulting in low accuracy in diagnosing mild hepatic steatosis and evaluating the severity of hepatic steatosis. In these cases, it is possible to diagnose and evaluate fatty liver disease by using a serologic marker using blood test results or by imaging an abdominal CT scan. In this regard, our study is the first study to present the usefulness of the HSI and the FLI in company with HU value on CT to overcome the limitations of liver ultrasound for the diagnosis and severity assessment of mild fatty liver disease based on the results of histological evaluation of hepatic steatosis. In addition, we first demonstrated that the HU values on CT could be useful in evaluating the degree of steatohepatitis in patients who have already been diagnosed with NAFLD through histological examination.

In patients with NAFLD, lifestyle modification and pharmacological intervention can improve liver histology, and thereby prognosis. Therefore, an accurate diagnosis of fatty liver is important in patients with suspected NAFLD. Many guidelines recommend ultrasound as the first-line tool for diagnosing NAFLD [2, 21]. However, ultrasound exhibits suboptimal performance in diagnosing mild hepatic steatosis. For example, Ahn et al. evaluated hepatic steatosis in living liver donors without evidence of fatty liver on ultrasonography, and have reported a high prevalence of mild hepatic steatosis of 39.6% in ultrasound-negative patients, suggesting that ultrasound cannot exclude mild hepatic steatosis [13]. Moreover, Tanaka et al. have reported that mild hepatic steatosis was diagnosed by biopsy in 28% of patients with elevation of serum ALT levels and normal hepatic ultrasound image [14]. In our study, in contrast to low diagnostic accuracy of ultrasound for diagnosing mild hepatic steatosis reported in the literature, the HSI and the FLI demonstrated high performance with AUROC of 0.810 and AUROC of 0.813, respectively, in diagnosing mild hepatic steatosis. The HSI is a non-invasive and non-imaging screening tool devised based on the Korean health check-up data [31]. When low and high cut-off values of the HSI were used to discriminate the presence or absence of NAFLD in patients included in the validation set of the original paper, a sensitivity of 93.1% and specificity of 93.1% were achieved. The FLI was devised based on Italian study which enrolled 280 persons with normal liver and 216 persons with hepatic disease. When high cut-off value of the FLI was used to discriminate the presence of NAFLD in patients, a positive predictive value of 99% and negative predictive value of 15% were achieved. The performance of the HSI and the FLI in diagnosing NAFLD has been validated in various studies. Lee et al. evaluated the performance of several screening scores for diagnosing NAFLD in patients who underwent a health checkup, and the HSI indicated a high AUROC of 0.86 [32]. Murayama et al. also evaluated the performance of the HSI, Zhejiang university index, and fatty liver index using ultrasound-diagnosed fatty liver as a reference standard, and the HSI and the FLI demonstrated good predictive ability with AUROC of 0.874 and 0.884, respertively [33]. However, many previous studies validated the performance of the HSI in diagnosing NAFLD using ultrasound as a reference standard. As mentioned above, because diagnosing mild hepatic steatosis using ultrasound may be inaccurate, in our judgment, these studies have some limitations because they are not based on histological evaluation. Our study was conducted defining fatty liver histologically as the presence of steatosis in > 5% of hepatocytes and in particular, we enrolled only patients with mild hepatic steatosis (steatosis in < 33% of hepatocytes). Therefore, we confirm good performance of the HSI and the FLI in diagnosing mild hepatic steatosis more objectively and strictly in our study than in previous studies. Considering the high sensitivity and specificity of HSI and the FLI for diagnosing mild hepatic steatosis observed here, additional tests to exclude mild hepatic steatosis might be beneficial for suspected NAFLD in patients with negative US findings but have the HSI of ≥ 36 or the FLI of ≥ 60.

Indeed, the liver HU value showed a low sensitivity in diagnosing mild hepatic steatosis in our study. The low sensitivity of CT in diagnosing mild hepatic steatosis has been reported in several previous studies [34, 35]. In particular, since the liver HU value may be affected by the reconstruction algorithm or the vendor of the CT scanner, the L–S HU value using spleen as an internal control is more commonly used for diagnosing fatty liver disease [35]. Therefore, when compared with the liver HU value in our study, the L–S HU value demonstrated significantly higher AUROC value and sensitivity in diagnosing mild hepatic steatosis, suggesting that the L–S HU value has advantage over liver HU value for detecting mild hepatic steatosis.

In addition to the limited diagnostic accuracy of ultrasound in detecting mild hepatic steatosis, the suboptimal performance to evaluate the degree of fatty liver, which may be due to the qualitative and subjective nature that causes inter-observer variability, is another disadvantage of ultrasound. Strauss et al. have reported that the inter-observer agreement for grading the severity of fatty liver using ultrasound was 47.0–63.7% [15]. Qayyum et al. have also reported that the correlation of ultrasound score with histological hepatic steatosis was low due to low inter-observer agreement for ultrasound [16]. In contrast to ultrasound, CT scan is not dependent on the operator. Our study revealed that the L–S HU value on CT could be better than ultrasound for quantifying and stratifying liver fat content, based on the results of histological evaluation as well as MRI-PDFF. Although proton magnetic resonance spectroscopy (^1^H-MRS) was recommended in clinical trials and experimental studies for the quantitative estimation of hepatic steatosis, it was not recommended in common clinical settings because of its high cost [21]. Recently, chemical-shift-encoded MRI (CSE-MRI) method has shown promising results, but accessibility to MRI is limited in various clinical settings, including primary care. Moreover, CT is almost routinely used clinically in patients with poor sonic view due to obesity or anatomical characteristics, although it is accompanied by elevated liver enzyme levels. Therefore, CT could have advantage over MRI for quantifying liver fat content in various particular clinical conditions, such as routine surveillance or opportunistic detection. Kramer et al. evaluated the diagnostic accuracy of various imaging methods in the quantification of hepatic steatosis using ^1^H-MRS as the reference standard, and reported an excellent correlation between HU value and ^1^H-MRS [36]. Another study demonstrated that CT-based liver fat quantification exhibited good correlation with MRI-PDFF measured by CSE-MRI [37]. Our results support the results of previous studies, using histological hepatic steatosis, as well as MRI-PDFF, as a reference standard. Consistent with the results of previous studies, our results demonstrate that CT-based liver fat quantification validated as a result of histological examination is a useful alternative to MRI-based liver fat quantification. Compared with previous studies, our study enrolled only patients with low-grade hepatic steatosis. Evaluation of the severity of fatty liver in these low-grade hepatic steatosis patients is of great clinical importance in terms of metabolic diseases. Several studies have emphasized that the degree of hepatic steatosis should be classified, even though in patients with mild hepatic steatosis. Li et al. have reported that patients with liver fat content > 10% had higher odds ratios of impaired glucose regulation than those with liver fat content < 10% [38]. Ducluzeau et al. have also reported that hepatic fat fraction between 5 and 10% confers the same risk of having the metabolic syndrome and that hepatic steatosis > 10% is associated with a very high probability of having the metabolic syndrome [39]. Therefore, if the L-S HU value presented by our study is used, it is expected to be helpful in classifying the severity of low-grade steatosis patients and to suggest a differentiated treatment strategy to the patients. In the future, further studies will be needed on whether the liver fat content measured using CT could predict the prognosis in patients with low-grade hepatic steatosis.

Our study also demonstrated that the HU values ​​were useful in evaluating the degree of intrahepatic inflammation in patients with biopsy-proven NAFLD. Hepatocyte injury and inflammation has been known to be the most important factors in the progression of fibrosis, and reducing intrahepatic inflammation has been known to be associated with improvement of fibrosis [26–29]. In particular, Brunt et al. found that an improvement in NAS of 2 points or more as well as resolution of NASH was most strongly associated with fibrosis improvement [30]. However, because biopsy is invasive, there is a limit to repeatedly performing liver biopsy to evaluating the improvement of NASH in patients who have been diagnosed with NASH through histological examination [40, 41]. Therefore, if there is a noninvasive method to evaluate the improvement of intrahepatic inflammation, it would be helpful to evaluate the effectiveness of treatment and to determine whether to continue current treatment or change to another treatment option. In this study, L-S HU values ​​discriminated patients with NAS of 3 or higher with a sensitivity of 92% and specificity of 90%, and patients with a NAS of 5 or higher with a sensitivity of 100% and specificity of 82% in patients with low-grade liver steatosis. Considering the high accuracy of the L-S HU value in evaluating the degree of intrahepatic inflammation observed in this study, we think that L-S HU value ​​could be used in determining the improvement of intrahepatic inflammation in patients with biopsy-proven NAFLD.

This study had several limitations. First, the number of enrolled patients was relatively small, which may have reduced the generalizability of the results. Second, the performance of t HU values, the HSI, and the FLI for diagnosing mild hepatic steatosis or grading the severity of hepatic steatosis could not be directly compared with that of ultrasound. Multicenter, large cohort prospective studies are required to overcome these limitations.

In conclusion, the HU values are feasible for quantifying and stratifying hepatic fat content and for evaluating the degree of intrahepatic inflammation, and the HSI and the FLI are also useful tools for diagnosing mild hepatic steatosis.

## Supplementary Information

**Additional file 1: Figure S1.** Comparison of the proportion of hepatic steatosis and NAS between patients with and without metabolic syndrome. (A) Flow chart showing enrollment of patients (B) Comparison of the proportion of patients with hepatic steatosis between patients with and without metabolic syndrome (c) Comparison of NAS between patients with and without metabolic syndrome.

**Additional file 2: Figure S2.** Flow chart showing enrollment of patients who underwent MRI-PDFF.

**Additional file 3: Figure S3.** The correlation between each index and MRI-PDFF. (A) Scatter plots showing the positive correlation between the hepatic steatosis index and MRI-PDFF (B) Scatter plots showing the negative correlation between liver HU value and MRI-PDFF (C) Scatter plots showing the negative correlation between liver HU value-spleen HU value and MRI-PDFF (D) Scatter plots showing the negative correlation between fatty liver index and MRI-PDFF.

**Additional file 4: Figure S4.** The comparison of each index according to steatosis grade group evaluated by MRI-PDFF and performance of each index in grading the severity of hepatic steatosis. (A) The comparison of hepatic steatosis index according to mild steatosis grade group. (B) The comparison of liver HU value according to mild steatosis grade group. (C) The comparison of liver HU value-spleen HU value according to mild steatosis grade group. (D) The comparison of fatty liver index according to mild steatosis grade group. Performance of hepatic steatosis index, liver HU value, liver HU value-spleen HU value and fatty liver index in grading the severity of hepatic steatosis was also shown. Mild G0 = Group consisting of patients with MRI-PDFF < 5%; mild G1 = Group consisting of patients with MRI-PDFF ≥ 5% and < 20%; mild G2 = Group consisting of patients with MRI-PDFF ≥ 20% and < 33%.

## Data Availability

The data that support the findings of this study are available from the corresponding authors upon reasonable request.

## Abbreviations

ALT: Alanine aminotransferase
AST: Aspartate aminotransferase
AUROC: Area under the receiver operating characteristic curve
BMI: Body mass index
CT: Computerized tomography
DM: Type 2 diabetes mellitus
^1^H-MRS: Proton magnetic resonance spectroscopy
HIS: Hepatic steatosis index
HU: Hounsfield unit
MRI: Magnetic resonance imaging
MRI-PDFF: Magnetic resonance imaging proton density fat fraction
NAFLD: Nonalcoholic fatty liver disease
NASH: Nonalcoholic steatohepatitis
TG: Triglyceride
