# Supplementary figures and images for: CT-based Hounsfield unit values reflect the degree of steatohepatitis in patients with low-grade fatty liver disease
Source: BMC Gastroenterol. 2023 Mar 17;23:77. doi: 10.1186/s12876-023-02717-3 (PMC10022198; doi:10.1186/s12876-023-02717-3)

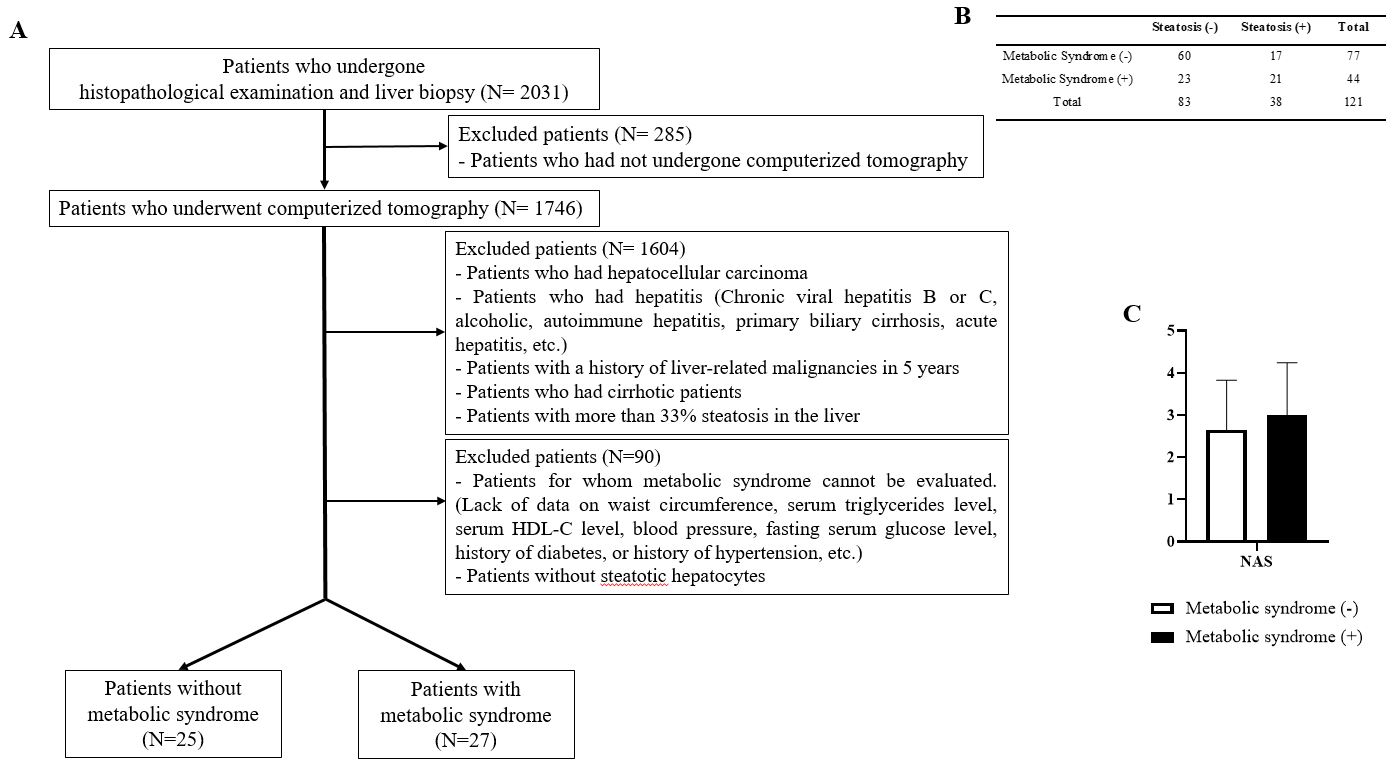

Supplement: Supplementary file 1 — Additional file 1: Figure S1. Comparison of the proportion of hepatic steatosis and NAS between patients with and without metabolic syndrome. (A) Flow chart showing enrollment of patients (B) Comparison of the proportion of patients with hepatic steatosis between patients with and without metabolic syndrome (c) Comparison of NAS between patients with and without metabolic syndrome. [file 12876_2023_2717_MOESM1_ESM.jpg]

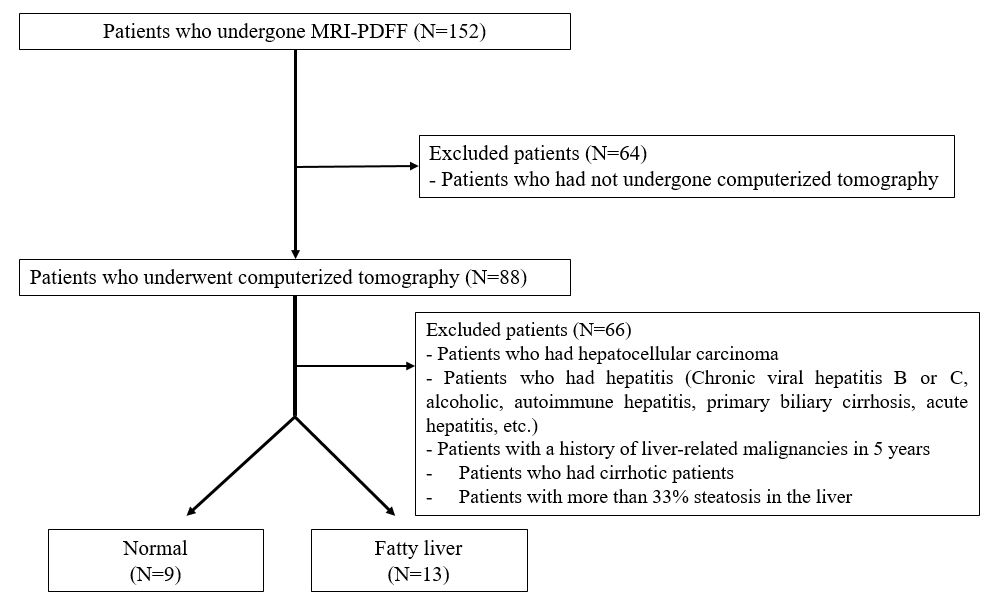

Supplement: Supplementary file 2 — Additional file 2: Figure S2. Flow chart showing enrollment of patients who underwent MRI-PDFF. [file 12876_2023_2717_MOESM2_ESM.jpg]

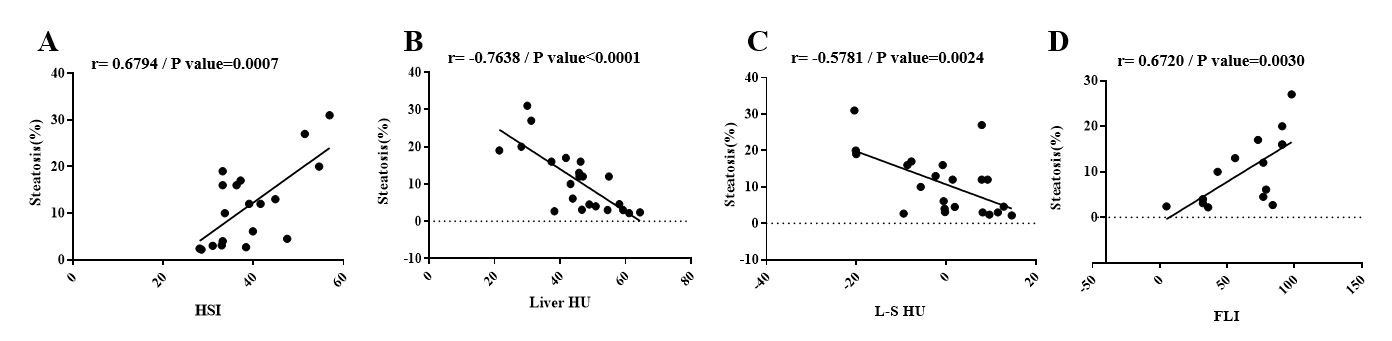

Supplement: Supplementary file 3 — Additional file 3: Figure S3. The correlation between each index and MRI-PDFF. (A) Scatter plots showing the positive correlation between the hepatic steatosis index and MRI-PDFF (B) Scatter plots showing the negative correlation between liver HU value and MRI-PDFF (C) Scatter plots showing the negative correlation between liver HU value-spleen HU value and MRI-PDFF (D) Scatter plots showing the negative correlation between fatty liver index and MRI-PDFF. [file 12876_2023_2717_MOESM3_ESM.jpg]

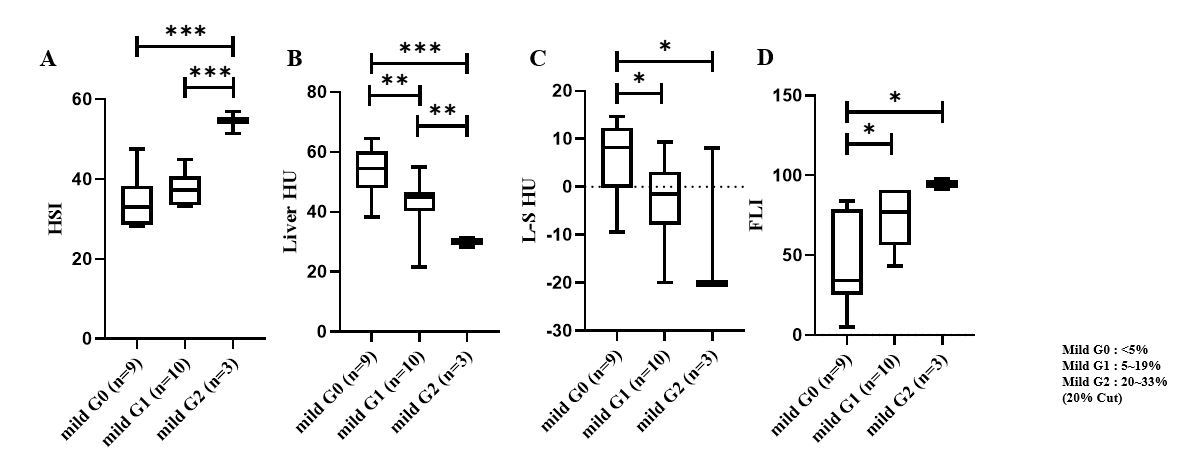

Supplement: Supplementary file 4 — Additional file 4: Figure S4. The comparison of each index according to steatosis grade group evaluated by MRI-PDFF and performance of each index in grading the severity of hepatic steatosis. (A) The comparison of hepatic steatosis index according to mild steatosis grade group. (B) The comparison of liver HU value according to mild steatosis grade group. (C) The comparison of liver HU value-spleen HU value according to mild steatosis grade group. (D) The comparison of fatty liver index according to mild steatosis grade group. Performance of hepatic steatosis index, liver HU value, liver HU value-spleen HU value and fatty liver index in grading the severity of hepatic steatosis was also shown. Mild G0 = Group consisting of patients with MRI-PDFF < 5%; mild G1 = Group consisting of patients with MRI-PDFF ≥ 5% and < 20%; mild G2 = Group consisting of patients with MRI-PDFF ≥ 20% and < 33%. [file 12876_2023_2717_MOESM4_ESM.jpg]
